# Crystal structure and Hirshfeld surface analysis of (*E*)-1-[2,2-di­chloro-1-(4-methyl­phen­yl)ethen­yl]-2-(4-meth­oxy­phen­yl)diazene

**DOI:** 10.1107/S2056989021008756

**Published:** 2021-08-27

**Authors:** Namiq Q. Shikhaliyev, Zeliha Atioğlu, Mehmet Akkurt, Ayten M. Qacar, Rizvan K. Askerov, Ajaya Bhattarai

**Affiliations:** a Organic Chemistry Department, Baku State University, Z. Khalilov str. 23, AZ 1148 Baku, Azerbaijan; b Department of Aircraft Electrics and Electronics, School of Applied Sciences, Cappadocia University, Mustafapaşa, 50420 Ürgüp, Nevşehir, Turkey; c Department of Physics, Faculty of Sciences, Erciyes University, 38039 Kayseri, Turkey; d Department of Chemistry, M.M.A.M.C (Tribhuvan University) Biratnagar, Nepal

**Keywords:** crystal structure, short C—H⋯Cl contacts, C—H⋯π inter­actions, van der Waals inter­actions, Hirshfeld surface analysis

## Abstract

The asymmetric unit of the title compound, C_16_H_14_Cl_2_N_2_O, comprises two similar mol­ecules, *A* and *B*, in which the dihedral angles between the two aromatic rings are 70.1 (3) and 73.2 (2)°, respectively. The crystal structure features short C—H⋯Cl and C—H⋯O contacts and C—H⋯π and van der Waals inter­actions. The title compound was refined as a two-component non-merohedral twin, BASF 0.1076 (5). The Hirshfeld surface analysis and two-dimensional fingerprint plots show that H⋯H (38.2% for mol­ecule *A*; 36.0% for mol­ecule *B*), Cl⋯H/H⋯Cl (24.6% for mol­ecule *A*; 26.7% for mol­ecule *B*) and C⋯H/H⋯C (20.0% for mol­ecule *A*; 20.2% for mol­ecule *B*) inter­actions are the most important contributors to the crystal packing.

## Abstract 2

Two similar mol­ecules make up the asymmetric unit of the title compound. The crystal structure features short C—H⋯Cl and C—H⋯O contacts and C—H⋯π and van der Waals inter­actions.

## Chemical context   

Azo dyes have found a wide range of applications, including as ligands, sensors, optical data storage, liquid crystals, non-linear optical materials, color-changing materials, mol­ecular switches, and dye-sensitized solar cells (Maharramov *et al.*, 2018 ▸; Mahmudov *et al.*, 2016 ▸; Viswanathan *et al.*, 2019 ▸). The functional properties of azo dyes are strongly dependent on the groups attached to the –N=N– synthon. Moreover, non-covalent bond donors or acceptors attached to *N*-donor azo/hydrazone ligands are of inter­est because of their high solubility in polar solvents, functional properties, photoactivity in the solid state, coordination ability, and high thermal and oxidative stability (Gurbanov *et al.*, 2020*a* ▸,*b* ▸; Kopylovich *et al.*, 2011 ▸; Mac Leod *et al.*, 2012 ▸; Mahmoudi *et al.*, 2017*a* ▸,*b* ▸, 2018*a* ▸,*b* ▸). The functionalization of *N*-donor ligands with –COOH or –SO_3_H groups can improve the catalytic activity of the corresponding metal complexes in oxidation and C—C coupling reactions (Gurbanov *et al.*, 2018 ▸; Ma *et al.*, 2017*a* ▸,*b* ▸, 2020 ▸, 2021 ▸; Mahmudov *et al.*, 2013 ▸; Mizar *et al.*, 2012 ▸; Shixaliyev *et al.*, 2014 ▸). Thus, in the current work we have synthesized a new azo dye, (*E*)-1-[2,2-di­chloro-1-(4-methyl­phen­yl)ethen­yl]-2-(4-meth­oxy­phen­yl)diazene, which displays multiple inter­molecular non-covalent inter­actions.

## Structural commentary   

**Figure 1:**
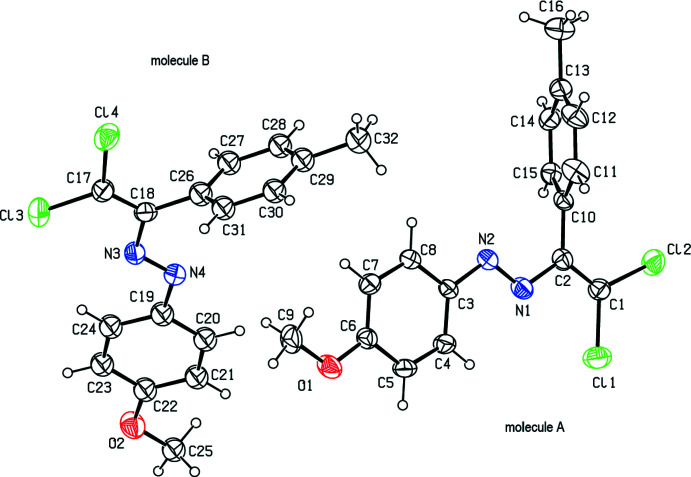
Mol­ecules *A* and *B* in the asymmetric unit with the atom-labeling scheme and ellipsoids drawn at the 30% probability level.

There are two comparable mol­ecules A (with Cl1) and B (with Cl3) in the asymmetric unit of the title compound (Fig. 1 ▸). The dihedral angles between the two aromatic rings (C3–C8/C10–C15 and C19–C24/C26–C31) in mol­ecules *A* and *B* are 70.1 (3) and 73.2 (2)°, respectively. In mol­ecule *A*, the N2/N1/C2/C1/Cl1/Cl2 moiety is approximately planar, with a maximum deviation of 0.110 (2) Å, and makes dihedral angles of 1.2 (2) and 71.3 (2)°, respectively, with the C3–C8 and C10–C15 rings. In mol­ecule *B*, the N4/N3/C18/C17/Cl3/Cl4 moiety is approximately planar with a maximum deviation of 0.046 (6) Å, and makes dihedral angles of 9.57 (18) and 75.94 (19)°, respectively, with the C19–C24 and C26–C31 rings.
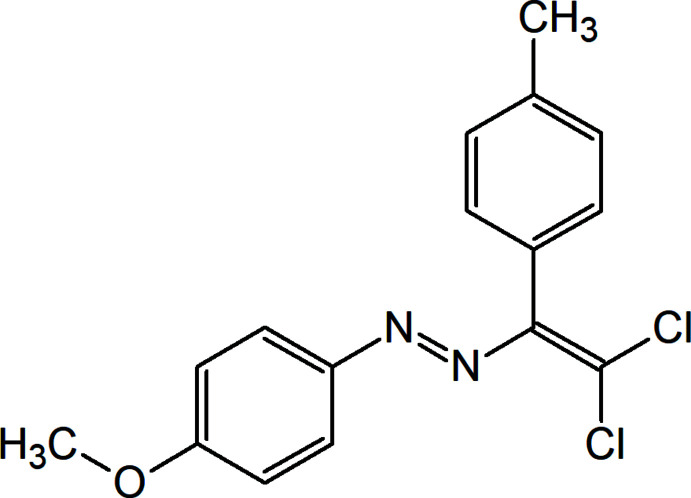


## Supra­molecular features   

**Figure 2:**
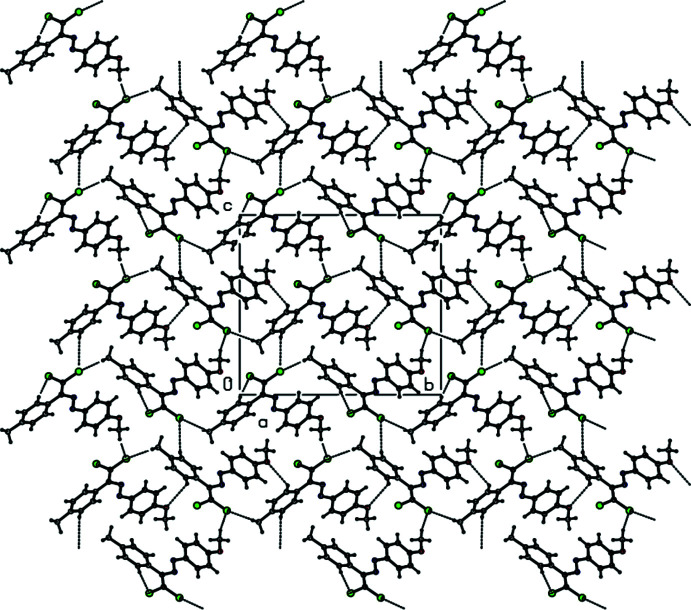
The crystal packing of the title compound viewed along the *b* axis, showing the C—H⋯Cl and C—H⋯O inter­actions as dashed lines.

**Figure 3:**
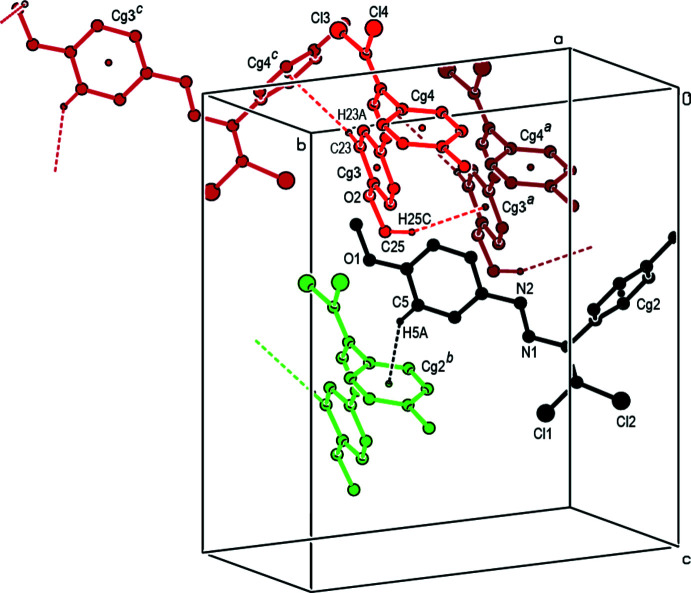
A general view of the C—H⋯π inter­actions in the title compound. [Symmetry codes: (*a*) −1 + *x*, *y*, *z*; (*b*) −*x*, 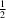 + *y*, 1 − *z*; (*c*) 1 − *x*, 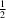 + *y*, −*z*].

In the crystal, no classical hydrogen bonds are observed. The mol­ecules are self-assembled *via* C—H⋯Cl short contacts, yielding supra­molecular chains along the *b*-axis direction. Adjacent chains are linked by C—H⋯O contacts, generating a two-dimensional array parallel to the *bc* plane (Table 1 ▸, Fig. 2 ▸). In addition, mol­ecules are connected by C—H⋯π inter­actions [Table 2 ▸, Fig. 3 ▸; C5—H5*A*⋯*Cg*2^i^, C23—H23*A*⋯*Cg*4^ii^ and C25—H25*C*⋯*Cg*3^ii^, where *Cg*2, *Cg*3 and *Cg*4 are the centroids of the benzene rings C10–C15 in mol­ecule *A*, and C19–C24 and C26–C31 in mol­ecule *B*, respectively]. The mol­ecular packing is further stabilized by van der Waals inter­actions.

## Hirshfeld surface analysis   

**Figure 4:**
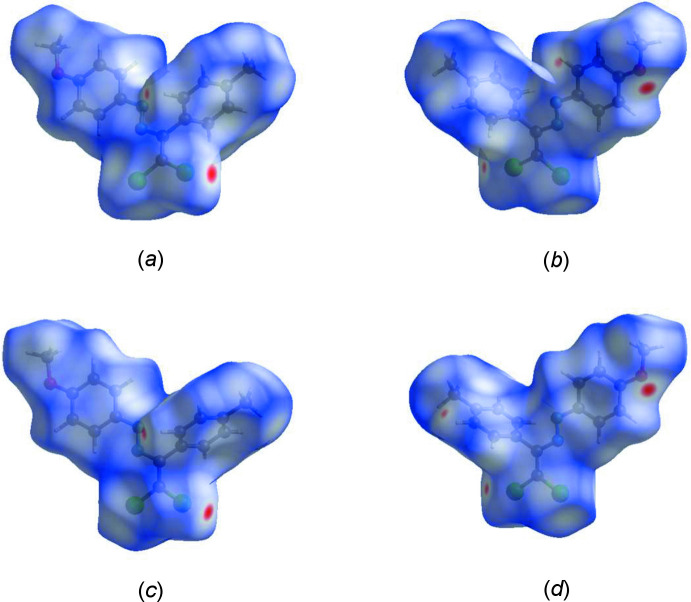
(*a*) Front and (*b*) back views of the Hirshfeld surface of mol­ecule *A*, and (*c*) front and (*d*) back views of the Hirshfeld surface of mol­ecule *B* plotted over *d*_norm_ in the ranges −0.1125 to 1.3054 and −0.1000 to 1.2923 a.u., respectively, for mol­ecules *A* and *B*.

**Figure 5:**
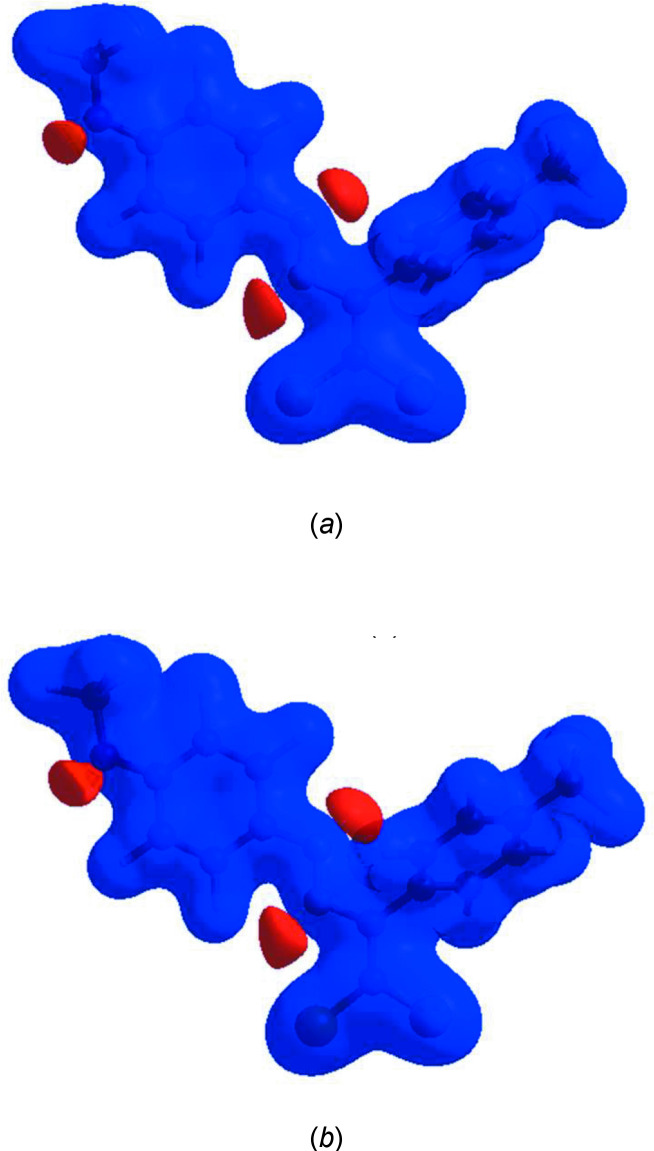
Views of the three-dimensional Hirshfeld surfaces of (*a*) mol­ecule *A* and (*b*) mol­ecule *B* plotted over electrostatic potential energy in the range −0.0500 to 0.0500 a.u. using the STO-3 G basis set at the Hartree–Fock level of theory. The hydrogen-bond donors and acceptors are shown as blue and red regions, respectively, around the atoms corresponding to positive and negative potentials.

To visualize the inter­molecular inter­actions in the title mol­ecule, *CrystalExplorer17* (Turner *et al.*, 2017 ▸) was used to generate Hirshfeld surfaces (McKinnon *et al.*, 2007 ▸) and their corresponding two-dimensional fingerprint plots (Spackman & McKinnon, 2002 ▸). In the Hirshfeld surfaces mapped over *d*_norm_ for mol­ecules *A* and *B* of the title compound (Fig. 4 ▸), the bright-red spots near atoms Cl1, Cl3, Cl4 and O1 indicate the short C—H⋯Cl and C—H⋯O contacts (Table 1 ▸). Other contacts are equal to or longer than the sum of van der Waals radii. The Hirshfeld surfaces for mol­ecules *A* and *B* mapped over electrostatic potential (Spackman *et al.*, 2008 ▸) are shown in Fig. 5 ▸. The positive electrostatic potential (blue regions) over the surface indicates hydrogen-donor potential, whereas the hydrogen-bond acceptors are represented by negative electrostatic potential (red regions).

**Figure 6:**
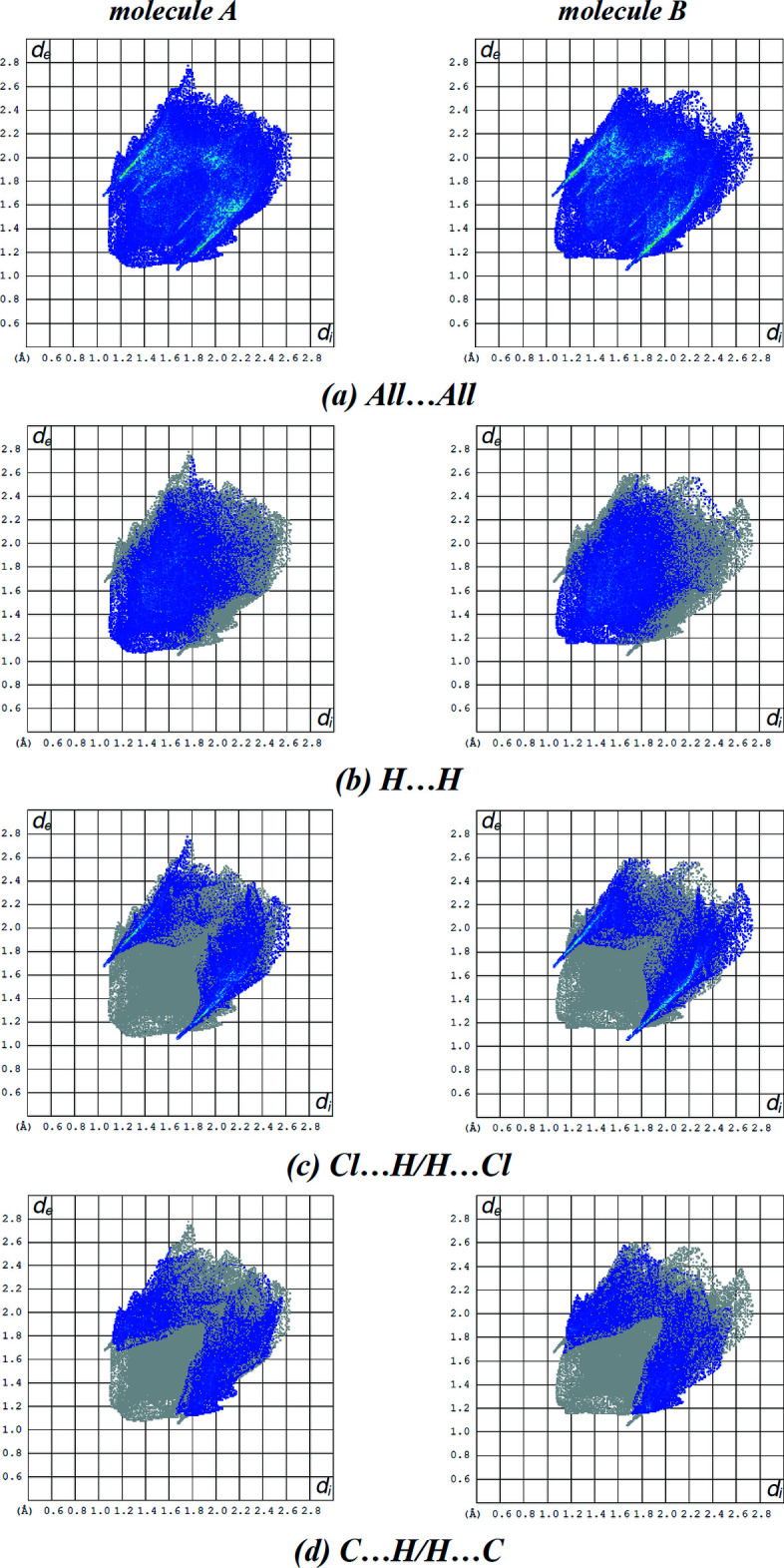
The full two-dimensional fingerprint plots for both mol­ecules *A* and *B* showing (*a*) all inter­actions, and delineated into (*b*) H⋯H, (*c*) Cl⋯H/H⋯Cl and (*d*) C⋯H/H⋯C inter­actions. The *d*_i_ and *d*_e_ values are the closest inter­nal and external distances (in Å) from given points on the Hirshfeld surface.

The overall two-dimensional fingerprint plot and those delineated into H⋯H, Cl⋯H/H⋯Cl and C⋯H/H⋯C contacts in mol­ecules *A* and *B* are illustrated in Fig. 6 ▸. The most important inter­action is H⋯H, contributing 38.2% for mol­ecule *A* and 36.0% for mol­ecule *B* to the overall crystal packing (Fig. 6 ▸ *b*). The Cl⋯H/H⋯Cl inter­actions appear as two symmetrical broad wings with *d*_e_ + *d*_i_ = 2.70 Å and contribute 24.6% to the Hirshfeld surface for mol­ecule *A*, and with *d*_e_ + *d*_i_ = 2.70 Å and contribute 26.7% to the Hirshfeld surface for mol­ecule *B* (Fig. 6 ▸ *c*). The pair of characteristic wings in the fingerprint plot delineated into H⋯C/C⋯H contacts (Fig. 6 ▸ *d*; 20.0% contribution for mol­ecule *A* and 20.2% contribution for mol­ecule *B*) have the tips at *d*_e_ + *d*_i_ = 2.80 Å for mol­ecule *A* and at *d*_e_ + *d*_i_ = 2.85 Å for mol­ecule *B*. The remaining contributions for both mol­ecules *A* and *B* are from N⋯H/H⋯N, O⋯H/H⋯O, N⋯C/C⋯N, Cl⋯O/O⋯Cl, Cl⋯C/C⋯Cl, C⋯C, Cl⋯N/N⋯Cl, O⋯C/C⋯O and Cl⋯Cl contacts, which are less than 4.6% and have a negligible effect on the packing. The percentage contributions of all inter­actions are listed in Table 3 ▸. The fact that the same inter­actions make different contributions to the HS for mol­ecules *A* and *B* can be attributed to the different mol­ecular environments of the *A* and *B* mol­ecules in the crystal structure.

## Database survey   

A search of the Cambridge Structural Database (CSD, Version 5.41, update of November 2019; Groom *et al.*, 2016 ▸) for the (*E*)-1-(2,2-di­chloro-1-phenyl­ethen­yl)-2-phenyl­diazene unit resulted in 27 hits. Eight compounds are closely related to the title compound, *viz*. 4-{2,2-di­chloro-1-[(3,5-di­methyl­phen­yl)diazen­yl]ethen­yl}-*N*,*N*-di­methyl­aniline (GUPHIL; Özkaraca *et al.*, 2020*a* ▸), 4-{2,2-di­chloro-1-[(4-fluoro­phen­yl)di­azen­yl]ethen­yl}-*N*,*N*-di­methyl­aniline (DUL­TAI; Özkaraca *et al.*, 2020*b* ▸), 1-(4-bromo­phen­yl)-2-[2,2-di­chloro-1-(4-nitro­phen­yl)ethen­yl]diazene (HONBOE; Akkurt *et al.*, 2019 ▸), 1-(4-chloro­phen­yl)-2-[2,2-di­chloro-1-(4-nitro­phen­yl)ethen­yl]di­azene (HONBUK; Akkurt *et al.*, 2019 ▸), 1-(4-chloro­phen­yl)-2-[2,2-di­chloro-1-(4-fluoro­phen­yl)ethen­yl]di­azene (HODQAV; Shikhaliyev *et al.*, 2019 ▸), 1-[2,2-di­chloro-1-(4-nitro­phen­yl)ethen­yl]-2-(4-fluoro­phen­yl)diazene (XIZ­REG; Atioğlu *et al.*, 2019 ▸), 1,1-[methyl­enebis(4,1-phenyl­ene)]bis­[(2,2-di­chloro-1-(4-nitro­phen­yl)ethen­yl]diazene (LEQXIR; Shikhaliyev *et al.*, 2018 ▸) and 1,1-[methyl­enebis(4,1-phenyl­ene)]bis­{[2,2-di­chloro-1-(4-chloro­phen­yl)ethen­yl]diazene} (LEQXOX; Shikhaliyev *et al.*, 2018 ▸).

In GUPHIL, the benzene rings subtend a dihedral angle of 77.07 (10)°. In the crystal, mol­ecules are associated into inversion dimers *via* short Cl⋯Cl contacts [3.3763 (9) Å]. In DULTAI, the dihedral angle between the two aromatic rings is 64.12 (14)°. The crystal structure is stabilized by a short C—H⋯Cl contact, C—Cl⋯π and van der Waals inter­actions. In HONBOE and HONBUK, the aromatic rings form dihedral angles of 60.9 (2) and 64.1 (2)°, respectively. In the crystals, mol­ecules are linked through weak *X*⋯*Cl* contacts (*X* = Br for HONBOE and Cl for HONBUK), C—H⋯Cl and C—Cl⋯π inter­actions into sheets parallel to the *ab* plane. Additional van der Waals inter­actions consolidate the three-dimensional packing. In HODQAV, the benzene rings make a dihedral angle of 56.13 (13)°. Mol­ecules are stacked in columns along the *a*-axis direction *via* weak C—H⋯Cl hydrogen bonds and face-to-face π–π stacking inter­actions. The crystal packing is further consolidated by short Cl⋯Cl contacts. In XIZREG, the benzene rings form a dihedral angle of 63.29 (8)° and the mol­ecules are linked by C—H⋯O hydrogen bonds into zigzag chains running along the *c*-axis direction. The crystal packing also features C—Cl⋯π, C—F⋯π and N—O⋯π inter­actions. In the crystals of LEQXIR and LEQXOX, the dihedral angles between the aromatic rings are 56.18 (12) and 60.31 (14)°, respectively. In LEQXIR, C—H⋯N and C—H⋯O hydrogen bonds and short C—Cl⋯O contacts occur and in LEQXOX, C—H⋯N and short Cl⋯Cl contacts are observed.

## Synthesis and crystallization   

The title compound was synthesized according to a reported method (Mukhtarova *et al.*, 2021 ▸; Shikhaliyev *et al.*, 2018 ▸, 2019 ▸). A 20 mL screw-neck vial was charged with DMSO (10 mL), (*Z*)-1-(4-meth­oxy­phen­yl)-2-(4-methyl­benzyl­id­ene)hydrazine (240 mg, 1 mmol), tetra­methyl­ethylenedi­amine (TMEDA; 295 mg, 2.5 mmol), CuCl (2 mg, 0.02 mmol) and CCl_4_ (20 mmol, 10 equiv). After 1–3 h (until TLC analysis showed complete consumption of the corresponding Schiff base), the reaction mixture was poured into a 0.01 *M* solution of HCl (100 mL, pH = 2–3), and extracted with di­chloro­methane (3 × 20 mL). The combined organic phase was washed with water (3 × 50 mL) and brine (30 mL), dried over anhydrous Na_2_SO_4_ and concentrated *in vacuo* by a rotary evaporator. The residue was purified by column chromatography on silica gel using appropriate mixtures of hexane and di­chloro­methane (3/1–1/1). Crystals suitable for X-ray analysis were obtained by slow evaporation of an ethanol solution. Colorless solid (65%); m.p. 355 K. Analysis calculated for C_16_H_14_Cl_2_N_2_O: C 59.83, H 4.39, N 8.72; found: C 59.78, H 4.32, N 8.69%. ^1^H NMR (300 MHz, Chloro­form-*d*) δ 7.79 (*d*, *J* = 9.0Hz, 2H, Ar), 7.26 (*d*, *J* = 8.0Hz, 2H, Ar), 7.10 (*d*, *J* = 8.0Hz, 2H, Ar), 6.95 (*d*, *J* = 9.0Hz, 2H, Ar), 3.88 (*s*, 3H, OCH_3_), 2.42 (*s*, 3H, CH_3_). ^13^C NMR (75 MHz, Chloro­form-*d*) δ 162.48, 148.12, 147.82, 138.47, 129.90, 129.76, 129.41, 128.85, 125.23, 114.14, 55.58 and 21.48. ESI–MS: *m*/*z*: 322.14 [*M* + H]^+^.

## Refinement details   

Crystal data, data collection and structure refinement details are summarized in Table 4 ▸. All H atoms were positioned geometrically and refined using a riding model, with C—H = 0.93 or 0.96 Å, and with *U*_iso_(H) = 1.2 or 1.5*U*_eq_(C). Owing to poor agreement between observed and calculated intensities, eight outliers (2 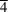 16, 2 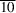 15, 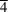 9 13, 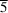 5 5, 1 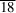 2, 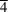 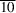 4, 4 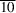 8 and 1 7 11) were omitted in the final cycles of refinement. The title compound was refined as a two-component non-merohedral twin, BASF 0.1076 (5).

## Supplementary Material

Crystal structure: contains datablock(s) I. DOI: 10.1107/S2056989021008756/zn2009sup1.cif

Structure factors: contains datablock(s) I. DOI: 10.1107/S2056989021008756/zn2009Isup2.hkl

Supporting information file. DOI: 10.1107/S2056989021008756/zn2009Isup3.cml

CCDC reference: 1984582

Additional supporting information:  crystallographic information; 3D view; checkCIF report

## Figures and Tables

**Table 1:** Hydrogen-bond geometry (Å, °) *Cg*2, *Cg*3 and *Cg*4 are the centroids of the benzene rings C10–C15 (in mol­ecule *A*) and C19–C24 and C26–C31 (in mol­ecule *B*), respectively.

*D*—H⋯*A*	*D*—H	H⋯*A*	*D*⋯*A*	*D*—H⋯*A*
C5—H5*A*⋯*Cg*2^i^	0.93	2.84	3.645 (8)	146
C23—H23*A*⋯*Cg*4^ii^	0.93	3.00	3.775 (5)	142
C25—H25*C*⋯*Cg*3^iii^	0.96	2.93	3.717 (7)	140

Symmetry codes: (i) -x, y+{\script{1\over 2}}, -z+1; (ii) -x+1, y+{\script{1\over 2}}, -z; (iii) x-1, y, z.

**Table 2:** Summary of short inter­atomic contacts (Å) in the title compound

Contact	Distance	Symmetry operation
Cl1⋯H16*C*	3.13	−1 − *x*, {1\over 2} + *y*, 1 − *z*
Cl1⋯H25*B*	3.06	−*x*, −{1\over 2} + *y*, 1 − *z*
O1⋯H11*A*	2.88	1 − *x*, {1\over 2} + *y*, 1 − *z*
H14*A*⋯Cl3	3.09	−*x*, −{1\over 2} + *y*, −*z*
Cl3⋯H32*A*	3.03	2 − *x*, {1\over 2} + *y*, −*z*
Cl4⋯H27*A*	2.88	1 + *x*, *y*, *z*

**Table 3:** Percentage contributions of inter­atomic contacts to the Hirshfeld surfaces for the mol­ecules A and B of the title compound in the asymmetric unit

Contact	Percentage contribution	
	mol­ecule *A*	mol­ecule *B*
H⋯H	38.2	36.0
Cl⋯H/H⋯Cl	24.6	26.7
C⋯H/H⋯C	20.0	20.2
N⋯H/H⋯N	4.5	4.6
O⋯H/H⋯O	3.2	3.1
N⋯C/C⋯N	3.1	3.2
Cl⋯O/O⋯Cl	2.0	2.3
Cl⋯C/C⋯Cl	1.8	1.7
C⋯C	1.3	1.2
Cl⋯N/N⋯Cl	1.1	0.9
O⋯C/C⋯O	0.2	0.3
Cl⋯Cl	0.1	0.1

**Table 4:** Experimental details

Crystal data	
Chemical formula	C_16_H_14_Cl_2_N_2_O
*M*_r_	321.19
Crystal system, space group	Monoclinic, *P*2_1_
Temperature (K)	296
*a*, *b*, *c* (Å)	5.5366 (3), 17.9208 (8), 16.2085 (8)
β (°)	99.173 (2)
*V* (Å^3^)	1587.65 (14)
*Z*	4
Radiation type	Mo *K*α
μ (mm^−1^)	0.41
Crystal size (mm)	0.24 × 0.19 × 0.10

Data collection	
Diffractometer	Bruker APEXII CCD
Absorption correction	Multi-scan (*SADABS*; Krause *et al.*, 2015 ▸)
*T*_min_, *T*_max_	0.675, 0.745
No. of measured, independent and observed [*I* > 2σ(*I*)] reflections	19301, 6444, 3820
*R*_int_	0.054
(sin θ/λ)_max_ (Å^−1^)	0.624

Refinement	
*R*[*F*^2^ > 2σ(*F* ^2^)], *wR*(*F* ^2^), *S*	0.059, 0.145, 1.01
No. of reflections	6444
No. of parameters	288
No. of restraints	1
H-atom treatment	H-atom parameters constrained
Δρ_max_, Δρ_min_ (e Å^−3^)	0.37, −0.32
Absolute structure	Refined as an inversion twin
Absolute structure parameter	0.11 (10)

Computer programs: *APEX2* (Bruker, 2007 ▸), *SAINT* (Bruker, 2007 ▸), *SHELXT2016/6* (Sheldrick, 2015*a* ▸), *SHELXL2016/6* (Sheldrick, 2015*b* ▸), *ORTEP-3 for Windows* (Farrugia, 2012 ▸) and *PLATON* (Spek, 2020 ▸).

## supplementary crystallographic information

### Crystal data

**Table:**

C_16_H_14_Cl_2_N_2_O	*F*(000) = 664
*M**_r_* = 321.19	*D*_x_ = 1.344 Mg m^−^^3^
Monoclinic, *P*2_1_	Mo *K*α radiation, λ = 0.71073 Å
*a* = 5.5366 (3) Å	Cell parameters from 3046 reflections
*b* = 17.9208 (8) Å	θ = 2.6–23.3°
*c* = 16.2085 (8) Å	µ = 0.41 mm^−^^1^
β = 99.173 (2)°	*T* = 296 K
*V* = 1587.65 (14) Å^3^	Prism, colourless
*Z* = 4	0.24 × 0.19 × 0.10 mm

### Data collection

**Table:**

Bruker APEXII CCD diffractometer	3820 reflections with *I* > 2σ(*I*)
φ and ω scans	*R*_int_ = 0.054
Absorption correction: multi-scan (SADABS; Krause *et al.*, 2015)	θ_max_ = 26.4°, θ_min_ = 1.7°
*T*_min_ = 0.675, *T*_max_ = 0.745	*h* = −6→6
19301 measured reflections	*k* = −22→22
6444 independent reflections	*l* = −20→20

### Refinement

**Table:**

Refinement on *F*^2^	Hydrogen site location: inferred from neighbouring sites
Least-squares matrix: full	H-atom parameters constrained
*R*[*F*^2^ > 2σ(*F*^2^)] = 0.059	*w* = 1/[σ^2^(*F*_o_^2^) + (0.0481*P*)^2^ + 0.5767*P*] where *P* = (*F*_o_^2^ + 2*F*_c_^2^)/3
*wR*(*F*^2^) = 0.145	(Δ/σ)_max_ < 0.001
*S* = 1.01	Δρ_max_ = 0.37 e Å^−^^3^
6444 reflections	Δρ_min_ = −0.32 e Å^−^^3^
288 parameters	Absolute structure: Refined as an inversion twin
1 restraint	Absolute structure parameter: 0.11 (10)

### Special details

**Table:**

Geometry. All esds (except the esd in the dihedral angle between two l.s. planes) are estimated using the full covariance matrix. The cell esds are taken into account individually in the estimation of esds in distances, angles and torsion angles; correlations between esds in cell parameters are only used when they are defined by crystal symmetry. An approximate (isotropic) treatment of cell esds is used for estimating esds involving l.s. planes.
Refinement. Refined as a 2-component inversion twin.

### Fractional atomic coordinates and isotropic or equivalent isotropic displacement parameters (Å^2^)

**Table:**

	*x*	*y*	*z*	*U*_iso_*/*U*_eq_	
Cl1	−0.2542 (4)	0.43617 (11)	0.64439 (13)	0.0796 (7)	
Cl2	−0.4487 (3)	0.28816 (11)	0.61549 (11)	0.0712 (6)	
O1	0.6974 (9)	0.6350 (3)	0.3727 (3)	0.0697 (14)	
N1	−0.0135 (10)	0.4111 (3)	0.5003 (4)	0.0535 (14)	
N2	0.0904 (10)	0.4034 (3)	0.4371 (3)	0.0489 (13)	
C1	−0.2741 (12)	0.3575 (4)	0.5831 (4)	0.0521 (16)	
C2	−0.1609 (11)	0.3501 (4)	0.5159 (4)	0.0488 (16)	
C3	0.2407 (11)	0.4641 (3)	0.4235 (4)	0.0467 (15)	
C4	0.2717 (12)	0.5288 (4)	0.4712 (4)	0.0562 (18)	
H4A	0.188500	0.534413	0.516384	0.067*	
C5	0.4230 (14)	0.5841 (4)	0.4525 (4)	0.0620 (19)	
H5A	0.441835	0.627135	0.484996	0.074*	
C6	0.5504 (12)	0.5769 (3)	0.3849 (4)	0.0489 (16)	
C7	0.5196 (12)	0.5138 (4)	0.3367 (4)	0.0530 (17)	
H7A	0.602750	0.508512	0.291554	0.064*	
C8	0.3636 (12)	0.4577 (4)	0.3556 (4)	0.0527 (17)	
H8A	0.341397	0.415233	0.322199	0.063*	
C9	0.8362 (14)	0.6303 (4)	0.3075 (5)	0.075 (2)	
H9A	0.958880	0.668681	0.314241	0.113*	
H9B	0.730904	0.636837	0.254871	0.113*	
H9C	0.913688	0.582341	0.308698	0.113*	
C10	−0.1948 (11)	0.2833 (3)	0.4611 (4)	0.0434 (14)	
C11	−0.0071 (12)	0.2325 (4)	0.4599 (5)	0.064 (2)	
H11A	0.141146	0.239262	0.495082	0.076*	
C12	−0.0390 (14)	0.1719 (4)	0.4065 (5)	0.067 (2)	
H12A	0.087072	0.137412	0.407601	0.080*	
C13	−0.2533 (14)	0.1614 (4)	0.3519 (4)	0.0596 (19)	
C14	−0.4408 (13)	0.2110 (4)	0.3547 (4)	0.0577 (18)	
H14A	−0.589706	0.203698	0.320052	0.069*	
C15	−0.4119 (11)	0.2715 (3)	0.4082 (4)	0.0464 (15)	
H15A	−0.540900	0.304650	0.408564	0.056*	
C16	−0.2854 (17)	0.0945 (5)	0.2927 (5)	0.091 (3)	
H16A	−0.140574	0.064261	0.302099	0.136*	
H16B	−0.312419	0.111859	0.235935	0.136*	
H16C	−0.423307	0.065509	0.302905	0.136*	
Cl3	0.7604 (5)	0.70082 (12)	−0.12906 (13)	0.0884 (8)	
Cl4	0.9416 (4)	0.55116 (12)	−0.10328 (12)	0.0782 (7)	
O2	−0.2060 (9)	0.9018 (3)	0.1289 (3)	0.0741 (15)	
N3	0.5200 (11)	0.6762 (3)	0.0147 (3)	0.0547 (14)	
N4	0.4238 (10)	0.6706 (3)	0.0789 (3)	0.0548 (14)	
C17	0.7780 (13)	0.6217 (4)	−0.0682 (4)	0.0569 (18)	
C18	0.6653 (12)	0.6147 (4)	−0.0012 (4)	0.0514 (17)	
C19	0.2694 (8)	0.7321 (2)	0.0907 (3)	0.0629 (6)	
C20	0.1554 (8)	0.7291 (2)	0.1610 (3)	0.0629 (6)	
H20A	0.186090	0.689285	0.198089	0.076*	
C21	−0.0044 (8)	0.7855 (3)	0.1760 (2)	0.0629 (6)	
H21A	−0.080656	0.783412	0.223126	0.076*	
C22	−0.0502 (8)	0.8449 (2)	0.1207 (3)	0.0629 (6)	
C23	0.0638 (9)	0.8479 (2)	0.0503 (3)	0.0629 (6)	
H23A	0.033133	0.887707	0.013237	0.076*	
C24	0.2236 (8)	0.7915 (2)	0.0353 (2)	0.0629 (6)	
H24A	0.299880	0.793580	−0.011802	0.076*	
C25	−0.3465 (13)	0.9007 (4)	0.1949 (4)	0.0629 (6)	
H25A	−0.457475	0.942189	0.189000	0.094*	
H25B	−0.239456	0.904082	0.247500	0.094*	
H25C	−0.437714	0.854952	0.192725	0.094*	
C26	0.6954 (9)	0.5466 (2)	0.0525 (3)	0.0629 (6)	
C27	0.5076 (7)	0.4946 (2)	0.0485 (3)	0.0629 (6)	
H27A	0.361256	0.502241	0.012536	0.076*	
C28	0.5386 (7)	0.4311 (2)	0.0983 (3)	0.0629 (6)	
H28A	0.412980	0.396257	0.095678	0.076*	
C29	0.7573 (8)	0.4196 (2)	0.1521 (3)	0.0629 (6)	
C30	0.9451 (7)	0.4716 (3)	0.1560 (3)	0.0629 (6)	
H30A	1.091434	0.463884	0.192023	0.076*	
C31	0.9141 (7)	0.5351 (2)	0.1062 (3)	0.0629 (6)	
H31A	1.039714	0.569868	0.108882	0.076*	
C32	0.7922 (16)	0.3491 (4)	0.2055 (5)	0.086 (3)	
H32A	0.926377	0.320655	0.190966	0.129*	
H32B	0.645678	0.319578	0.195512	0.129*	
H32C	0.826528	0.362584	0.263450	0.129*	

### Atomic displacement parameters (Å^2^)

**Table:**

	*U*^11^	*U*^22^	*U*^33^	*U*^12^	*U*^13^	*U*^23^
Cl1	0.0944 (16)	0.0730 (15)	0.0764 (13)	−0.0152 (12)	0.0289 (12)	−0.0239 (11)
Cl2	0.0750 (13)	0.0808 (15)	0.0611 (11)	−0.0262 (11)	0.0212 (10)	−0.0003 (10)
O1	0.080 (4)	0.051 (3)	0.088 (4)	−0.016 (3)	0.042 (3)	−0.005 (3)
N1	0.056 (4)	0.044 (3)	0.062 (3)	−0.005 (3)	0.013 (3)	0.000 (3)
N2	0.056 (3)	0.040 (3)	0.051 (3)	0.002 (3)	0.009 (3)	0.001 (2)
C1	0.051 (4)	0.053 (4)	0.052 (4)	−0.007 (3)	0.008 (3)	−0.003 (3)
C2	0.049 (4)	0.046 (4)	0.053 (4)	−0.003 (3)	0.011 (3)	0.003 (3)
C3	0.058 (4)	0.034 (3)	0.050 (4)	−0.008 (3)	0.016 (3)	−0.001 (3)
C4	0.069 (5)	0.050 (4)	0.057 (4)	−0.015 (4)	0.031 (4)	−0.012 (3)
C5	0.084 (5)	0.048 (4)	0.060 (4)	−0.014 (4)	0.031 (4)	−0.018 (3)
C6	0.053 (4)	0.042 (4)	0.054 (4)	0.004 (3)	0.016 (3)	0.002 (3)
C7	0.060 (4)	0.051 (4)	0.053 (4)	0.001 (3)	0.024 (3)	−0.005 (3)
C8	0.069 (4)	0.039 (4)	0.053 (4)	−0.005 (3)	0.018 (3)	−0.004 (3)
C9	0.083 (6)	0.072 (5)	0.080 (5)	−0.010 (4)	0.041 (5)	0.015 (4)
C10	0.045 (3)	0.036 (3)	0.051 (3)	−0.004 (3)	0.013 (3)	0.001 (3)
C11	0.042 (4)	0.054 (5)	0.094 (6)	0.003 (3)	0.007 (4)	−0.002 (4)
C12	0.061 (5)	0.045 (4)	0.098 (6)	0.009 (4)	0.021 (4)	−0.001 (4)
C13	0.073 (5)	0.049 (4)	0.062 (4)	0.002 (4)	0.027 (4)	−0.002 (3)
C14	0.062 (5)	0.065 (5)	0.046 (4)	−0.004 (4)	0.008 (3)	−0.001 (3)
C15	0.043 (4)	0.047 (4)	0.049 (3)	0.008 (3)	0.007 (3)	0.005 (3)
C16	0.123 (8)	0.062 (5)	0.091 (6)	−0.001 (5)	0.027 (6)	−0.021 (5)
Cl3	0.125 (2)	0.0759 (16)	0.0683 (13)	0.0050 (13)	0.0272 (13)	0.0208 (11)
Cl4	0.0905 (17)	0.0858 (16)	0.0647 (12)	0.0200 (12)	0.0323 (11)	0.0026 (11)
O2	0.080 (4)	0.061 (3)	0.087 (4)	0.017 (3)	0.032 (3)	0.010 (3)
N3	0.065 (4)	0.048 (4)	0.054 (3)	0.004 (3)	0.019 (3)	0.005 (3)
N4	0.066 (4)	0.048 (3)	0.052 (3)	0.000 (3)	0.013 (3)	−0.001 (3)
C17	0.063 (5)	0.057 (4)	0.052 (4)	0.005 (4)	0.011 (4)	0.001 (3)
C18	0.057 (4)	0.047 (4)	0.049 (4)	0.003 (3)	0.005 (3)	−0.008 (3)
C19	0.0714 (16)	0.0556 (14)	0.0645 (13)	0.0030 (11)	0.0190 (11)	0.0027 (10)
C20	0.0714 (16)	0.0556 (14)	0.0645 (13)	0.0030 (11)	0.0190 (11)	0.0027 (10)
C21	0.0714 (16)	0.0556 (14)	0.0645 (13)	0.0030 (11)	0.0190 (11)	0.0027 (10)
C22	0.0714 (16)	0.0556 (14)	0.0645 (13)	0.0030 (11)	0.0190 (11)	0.0027 (10)
C23	0.0714 (16)	0.0556 (14)	0.0645 (13)	0.0030 (11)	0.0190 (11)	0.0027 (10)
C24	0.0714 (16)	0.0556 (14)	0.0645 (13)	0.0030 (11)	0.0190 (11)	0.0027 (10)
C25	0.0714 (16)	0.0556 (14)	0.0645 (13)	0.0030 (11)	0.0190 (11)	0.0027 (10)
C26	0.0714 (16)	0.0556 (14)	0.0645 (13)	0.0030 (11)	0.0190 (11)	0.0027 (10)
C27	0.0714 (16)	0.0556 (14)	0.0645 (13)	0.0030 (11)	0.0190 (11)	0.0027 (10)
C28	0.0714 (16)	0.0556 (14)	0.0645 (13)	0.0030 (11)	0.0190 (11)	0.0027 (10)
C29	0.0714 (16)	0.0556 (14)	0.0645 (13)	0.0030 (11)	0.0190 (11)	0.0027 (10)
C30	0.0714 (16)	0.0556 (14)	0.0645 (13)	0.0030 (11)	0.0190 (11)	0.0027 (10)
C31	0.0714 (16)	0.0556 (14)	0.0645 (13)	0.0030 (11)	0.0190 (11)	0.0027 (10)
C32	0.120 (8)	0.064 (6)	0.079 (6)	0.005 (5)	0.030 (5)	0.004 (4)

### Geometric parameters (Å, º)

**Table:**

Cl1—C1	1.717 (7)	Cl3—C17	1.721 (7)
Cl2—C1	1.708 (7)	Cl4—C17	1.704 (7)
O1—C6	1.355 (7)	O2—C22	1.356 (5)
O1—C9	1.406 (8)	O2—C25	1.419 (8)
N1—N2	1.260 (7)	N3—N4	1.247 (7)
N1—C2	1.411 (8)	N3—C18	1.413 (8)
N2—C3	1.408 (7)	N4—C19	1.426 (6)
C1—C2	1.347 (9)	C17—C18	1.342 (9)
C2—C10	1.486 (8)	C18—C26	1.493 (7)
C3—C8	1.388 (8)	C19—C20	1.3900
C3—C4	1.390 (8)	C19—C24	1.3900
C4—C5	1.363 (9)	C20—C21	1.3900
C4—H4A	0.9300	C20—H20A	0.9300
C5—C6	1.401 (9)	C21—C22	1.3900
C5—H5A	0.9300	C21—H21A	0.9300
C6—C7	1.369 (8)	C22—C23	1.3900
C7—C8	1.391 (8)	C23—C24	1.3900
C7—H7A	0.9300	C23—H23A	0.9300
C8—H8A	0.9300	C24—H24A	0.9300
C9—H9A	0.9600	C25—H25A	0.9600
C9—H9B	0.9600	C25—H25B	0.9600
C9—H9C	0.9600	C25—H25C	0.9600
C10—C15	1.376 (8)	C26—C27	1.3900
C10—C11	1.384 (8)	C26—C31	1.3900
C11—C12	1.383 (9)	C27—C28	1.3900
C11—H11A	0.9300	C27—H27A	0.9300
C12—C13	1.375 (10)	C28—C29	1.3900
C12—H12A	0.9300	C28—H28A	0.9300
C13—C14	1.373 (9)	C29—C30	1.3900
C13—C16	1.527 (10)	C29—C32	1.526 (8)
C14—C15	1.382 (8)	C30—C31	1.3900
C14—H14A	0.9300	C30—H30A	0.9300
C15—H15A	0.9300	C31—H31A	0.9300
C16—H16A	0.9600	C32—H32A	0.9600
C16—H16B	0.9600	C32—H32B	0.9600
C16—H16C	0.9600	C32—H32C	0.9600
			
C6—O1—C9	118.5 (6)	C22—O2—C25	119.8 (5)
N2—N1—C2	114.4 (5)	N4—N3—C18	114.9 (5)
N1—N2—C3	113.6 (5)	N3—N4—C19	113.2 (5)
C2—C1—Cl2	122.4 (5)	C18—C17—Cl4	122.7 (6)
C2—C1—Cl1	123.6 (5)	C18—C17—Cl3	123.4 (6)
Cl2—C1—Cl1	114.0 (4)	Cl4—C17—Cl3	113.9 (4)
C1—C2—N1	115.2 (6)	C17—C18—N3	115.2 (6)
C1—C2—C10	122.2 (6)	C17—C18—C26	121.7 (6)
N1—C2—C10	122.6 (5)	N3—C18—C26	123.1 (5)
C8—C3—C4	118.6 (6)	C20—C19—C24	120.0
C8—C3—N2	115.9 (6)	C20—C19—N4	116.1 (4)
C4—C3—N2	125.5 (5)	C24—C19—N4	123.9 (4)
C5—C4—C3	120.5 (6)	C19—C20—C21	120.0
C5—C4—H4A	119.7	C19—C20—H20A	120.0
C3—C4—H4A	119.7	C21—C20—H20A	120.0
C4—C5—C6	120.8 (6)	C22—C21—C20	120.0
C4—C5—H5A	119.6	C22—C21—H21A	120.0
C6—C5—H5A	119.6	C20—C21—H21A	120.0
O1—C6—C7	125.1 (6)	O2—C22—C21	124.5 (4)
O1—C6—C5	115.6 (6)	O2—C22—C23	115.5 (4)
C7—C6—C5	119.3 (6)	C21—C22—C23	120.0
C6—C7—C8	119.8 (6)	C24—C23—C22	120.0
C6—C7—H7A	120.1	C24—C23—H23A	120.0
C8—C7—H7A	120.1	C22—C23—H23A	120.0
C3—C8—C7	120.9 (6)	C23—C24—C19	120.0
C3—C8—H8A	119.5	C23—C24—H24A	120.0
C7—C8—H8A	119.5	C19—C24—H24A	120.0
O1—C9—H9A	109.5	O2—C25—H25A	109.5
O1—C9—H9B	109.5	O2—C25—H25B	109.5
H9A—C9—H9B	109.5	H25A—C25—H25B	109.5
O1—C9—H9C	109.5	O2—C25—H25C	109.5
H9A—C9—H9C	109.5	H25A—C25—H25C	109.5
H9B—C9—H9C	109.5	H25B—C25—H25C	109.5
C15—C10—C11	118.2 (6)	C27—C26—C31	120.0
C15—C10—C2	120.7 (6)	C27—C26—C18	120.5 (4)
C11—C10—C2	121.0 (6)	C31—C26—C18	119.5 (4)
C12—C11—C10	120.3 (7)	C28—C27—C26	120.0
C12—C11—H11A	119.9	C28—C27—H27A	120.0
C10—C11—H11A	119.9	C26—C27—H27A	120.0
C13—C12—C11	121.4 (7)	C27—C28—C29	120.0
C13—C12—H12A	119.3	C27—C28—H28A	120.0
C11—C12—H12A	119.3	C29—C28—H28A	120.0
C14—C13—C12	118.0 (7)	C30—C29—C28	120.0
C14—C13—C16	121.0 (7)	C30—C29—C32	120.2 (5)
C12—C13—C16	120.9 (7)	C28—C29—C32	119.8 (5)
C13—C14—C15	121.1 (7)	C29—C30—C31	120.0
C13—C14—H14A	119.5	C29—C30—H30A	120.0
C15—C14—H14A	119.5	C31—C30—H30A	120.0
C10—C15—C14	120.9 (6)	C30—C31—C26	120.0
C10—C15—H15A	119.6	C30—C31—H31A	120.0
C14—C15—H15A	119.6	C26—C31—H31A	120.0
C13—C16—H16A	109.5	C29—C32—H32A	109.5
C13—C16—H16B	109.5	C29—C32—H32B	109.5
H16A—C16—H16B	109.5	H32A—C32—H32B	109.5
C13—C16—H16C	109.5	C29—C32—H32C	109.5
H16A—C16—H16C	109.5	H32A—C32—H32C	109.5
H16B—C16—H16C	109.5	H32B—C32—H32C	109.5
			
C2—N1—N2—C3	−178.8 (5)	C18—N3—N4—C19	−177.2 (5)
Cl2—C1—C2—N1	−176.8 (5)	Cl4—C17—C18—N3	−174.6 (5)
Cl1—C1—C2—N1	2.5 (9)	Cl3—C17—C18—N3	2.6 (9)
Cl2—C1—C2—C10	4.8 (9)	Cl4—C17—C18—C26	6.0 (10)
Cl1—C1—C2—C10	−175.9 (5)	Cl3—C17—C18—C26	−176.8 (5)
N2—N1—C2—C1	−179.8 (6)	N4—N3—C18—C17	−177.2 (6)
N2—N1—C2—C10	−1.4 (9)	N4—N3—C18—C26	2.2 (9)
N1—N2—C3—C8	178.7 (6)	N3—N4—C19—C20	179.7 (4)
N1—N2—C3—C4	−2.2 (9)	N3—N4—C19—C24	1.8 (7)
C8—C3—C4—C5	−1.1 (11)	C24—C19—C20—C21	0.0
N2—C3—C4—C5	179.7 (7)	N4—C19—C20—C21	−178.0 (5)
C3—C4—C5—C6	−0.1 (11)	C19—C20—C21—C22	0.0
C9—O1—C6—C7	−2.0 (10)	C25—O2—C22—C21	−4.0 (8)
C9—O1—C6—C5	178.1 (6)	C25—O2—C22—C23	174.8 (5)
C4—C5—C6—O1	−179.3 (7)	C20—C21—C22—O2	178.8 (5)
C4—C5—C6—C7	0.8 (11)	C20—C21—C22—C23	0.0
O1—C6—C7—C8	179.9 (6)	O2—C22—C23—C24	−178.9 (5)
C5—C6—C7—C8	−0.3 (10)	C21—C22—C23—C24	0.0
C4—C3—C8—C7	1.6 (10)	C22—C23—C24—C19	0.0
N2—C3—C8—C7	−179.2 (6)	C20—C19—C24—C23	0.0
C6—C7—C8—C3	−0.9 (10)	N4—C19—C24—C23	177.8 (5)
C1—C2—C10—C15	71.9 (8)	C17—C18—C26—C27	−105.8 (6)
N1—C2—C10—C15	−106.4 (7)	N3—C18—C26—C27	74.8 (7)
C1—C2—C10—C11	−110.1 (7)	C17—C18—C26—C31	73.7 (7)
N1—C2—C10—C11	71.6 (8)	N3—C18—C26—C31	−105.7 (6)
C15—C10—C11—C12	0.2 (10)	C31—C26—C27—C28	0.0
C2—C10—C11—C12	−177.9 (6)	C18—C26—C27—C28	179.5 (5)
C10—C11—C12—C13	1.9 (11)	C26—C27—C28—C29	0.0
C11—C12—C13—C14	−3.4 (11)	C27—C28—C29—C30	0.0
C11—C12—C13—C16	179.4 (7)	C27—C28—C29—C32	−178.9 (5)
C12—C13—C14—C15	2.8 (10)	C28—C29—C30—C31	0.0
C16—C13—C14—C15	180.0 (6)	C32—C29—C30—C31	178.9 (5)
C11—C10—C15—C14	−0.7 (9)	C29—C30—C31—C26	0.0
C2—C10—C15—C14	177.4 (5)	C27—C26—C31—C30	0.0
C13—C14—C15—C10	−0.8 (10)	C18—C26—C31—C30	−179.5 (5)

### Hydrogen-bond geometry (Å, º)

*Cg*2, *Cg*3 and *Cg*4 are the centroids of the benzene rings C10–C15 (in molecule *A*) and C19–C24 and C26–C31 (in molecule *B*), respectively.

**Table:**

*D*—H···*A*	*D*—H	H···*A*	*D*···*A*	*D*—H···*A*
C5—H5*A*···*Cg*2^i^	0.93	2.84	3.645 (8)	146
C23—H23*A*···*Cg*4^ii^	0.93	3.00	3.775 (5)	142
C25—H25*C*···*Cg*3^iii^	0.96	2.93	3.717 (7)	140

Symmetry codes: (i) −*x*, *y*+1/2, −*z*+1; (ii) −*x*+1, *y*+1/2, −*z*; (iii) *x*−1, *y*, *z*.
